# The Genomic Architecture of Bladder Exstrophy Epispadias Complex

**DOI:** 10.3390/genes12081149

**Published:** 2021-07-28

**Authors:** Glenda M. Beaman, Raimondo M. Cervellione, David Keene, Heiko Reutter, William G. Newman

**Affiliations:** 1 Division of Evolution and Genomic Sciences, Faculty of Biology, School of Biological Sciences, Medicine and Health, University of Manchester, Manchester M13 9PL, UK; glenda.beaman@manchester.ac.uk; 2 Manchester Centre for Genomic Medicine, Manchester University NHS Foundation Trust, Manchester M13 9WL, UK; 3 Royal Manchester Children’s Hospital, Manchester University NHS Foundation Trust, Manchester M13 9WL, UK; raimondo.cervellione@mft.nhs.uk (R.M.C.); David.Keene@mft.nhs.uk (D.K.); 4 Department of Neonatology and Paediatric Intensive Care, University Hospital Erlangen, 91054 Erlangen, Germany; Heiko.Reutter@ukbonn.de

**Keywords:** BEEC, bladder exstrophy, epispadias, cloacal exstrophy

## Abstract

The bladder exstrophy–epispadias complex (BEEC) is an abdominal midline malformation comprising a spectrum of congenital genitourinary abnormalities of the abdominal wall, pelvis, urinary tract, genitalia, anus, and spine. The vast majority of BEEC cases are classified as non-syndromic and the etiology of this malformation is still unknown. This review presents the current knowledge on this multifactorial disorder, including phenotypic and anatomical characterization, epidemiology, proposed developmental mechanisms, existing animal models, and implicated genetic and environmental components.

## 1. Introduction

Congenital anomalies of the lower urinary tract (CALUT) are a group of birth defects of the ureter, bladder, and urethra, which includes bladder exstrophy–epispadias complex (BEEC, MIM #600057). BEEC is an abdominal midline malformation comprising a spectrum of congenital genitourinary abnormalities of the abdominal wall, pelvis, urinary tract, genitalia, anus, and spine [1]. The severity of BEEC ranges from epispadias (E), representing the mildest form to include classic bladder exstrophy (CBE), and extending to cloacal exstrophy (CE), the latter complex—previously referred to as OEIS (omphalocele, exstrophy, imperforate anus, and spinal defects)—being the most severe [1,2]. BEEC is further subdivided into “classic/typical” forms (E, CBE, and CE) and “atypical” forms (duplicated exstrophy, covered exstrophy, and pseudo-exstrophy) [1,3]. In the majority of cases, BEEC is non-syndromic (that is, it is not associated with other congenital birth malformations). The etiology of this malformation is still unknown. Theories have proposed an abnormal overdevelopment of the cloacal membrane preventing medial migration of mesenchyme between the ectodermal and endodermal layers of the lower abdominal wall, resulting in abnormal development of the lower abdominal wall [4] or the involvement of cloacal membrane and mesenchymal tissues during their defective embryogenesis [5,6].

## 2. Epidemiology

Epispadias is rare, with incidences of one in 101,000 live births in males and one in 1,300,000 in females [7,8]. CBE has an incidence of one in 46,000 live births and is nearly twice as common in males as females [8,9]. CE is less common, with an incidence of one in 317,000 live births and with similar rates in males and females [8,10].

## 3. Clinical Description

### 3.1. Epispadias

Epispadias is generally diagnosed at birth, although its presentation is dependent on severity and sex. It consists of a dorsal located ectopic urethral meatus as a result of non-closure of the urethral plate during embryological development [3]. In both sexes, epispadias affects the genitalia and the pubic symphysis. The pubic symphysis is diastatic, with divergent distal rectus abdominis muscles, and is either closed or has a noticeable minor gap [1]. Urinary incontinence is the main clinical symptom, dependent upon the involvement of the urinary sphincter [3]. In males, an ectopic meatus may be located on the dorsal aspect of the penile shaft, glans, or the penopubic junction. Generally, the phallus is broad and short with a dorsal chordee and an absent dorsal foreskin [3]. In females, epispadias is distinguished by the degree of severity [3]. In less severe forms, the urethral meatus may appear patulous or have a uniformly bifid clitoris with superiorly divergent labia. In the most severe forms, the entire urethra is affected and involves the bladder neck displaying bladder mucosal prolapse [3].

### 3.2. Classic Bladder Exstrophy

**Figure 1:**
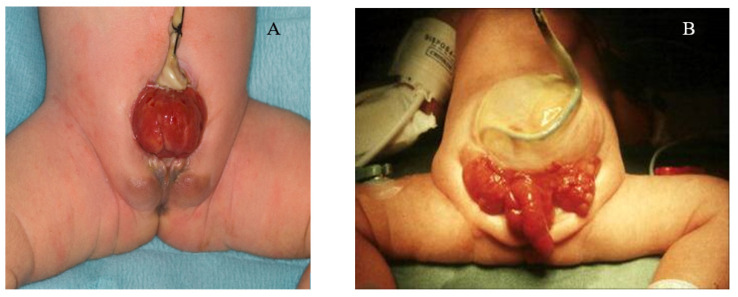
(**A**) Female with classic bladder exstrophy; (**B**) male with cloacal exstrophy.

CBE presents as a protrusion of the urinary bladder through a defect on the infraumbilical abdomen, in association with a diastasis of the pubic symphysis with distally divergent rectus abdominis muscles (Figure 1A) [3]. Pubic separation results in characteristic genital and pelvic abnormalities [7]. In both sexes the umbilicus is lower than normal and the distance between the umbilicus and the anus is shortened. The exstrophic bladder template is visible as a patch of reddened mucosa from which urine will drip from the urethral orifices on the bladder template, and in some cases, mucosal polyps may develop [3,7]. In males, CBE generally presents as an open urethral plate that covers the entire dorsum of the penis from the open bladder to the glandular grove. The penis appears shorter in length and dorsally curved. Inguinal hernias are extremely common [3,7]. In females, diastasis of the pubic symphysis results in the absence of a mons pubis with a bifid clitoris and displacement of the labia [7].

### 3.3. Cloacal Exstrophy

CE is a major birth defect in which the bladder is widely open on the infraumbilical abdominal wall and is separated into two distinct halves. It is often associated with omphalocele, separated pubic bones, short-gut syndrome, and other malformations, including talipes and spina bifida (Figure 1B) [1,11]. Typically, a foreshortened hindgut or cecum ends between two exstrophied hemi-bladders and the orifice of the terminal ileum is located at the everted cecum [3]. In males, the phallus is small and bifid with a hemi-glans caudal to each hemi-bladder [12,13]. In females, the uterus is generally bicornuate, with varying degrees of Müllerian duplication, two hemi-vaginas, and a bifid clitoris [12,13].

## 4. Evidence of a Genetic Basis to BEEC

The vast majority of BEEC cases are non-syndromic, however, a number of cases have been reported whereby BEEC has also been associated with various other syndromes, malformations, and congenital diseases (Table 1). There are a number of reported cases of OEIS (CE) with multiple cardiac malformations [14,15,16]. A population study undertaken by Kallen et al., 2000 [17] of 5260 infants with multiple malformations identified 194 OEIS cases; however, no association with cardiac defects was detected.

The majority of individuals affected by BEEC have no positive family history of BEEC. However, even though familial occurrence is rare, 30 multiplex families have been described [18,19,20,21]. A number of these appear to follow a Mendelian mode of inheritance. However, in the majority of affected individuals, the genetic basis of BEEC is consistent with a multifactorial etiology [22]. In the majority of multiplex families, only two members are affected. Two families have been reported with three affected members, including males and females with differing degrees of BEEC severity [18]. Reutter et al., 2003 [19] described a unique Moroccan family of three males (two cousins and a maternal uncle) being affected with CBE. In these rare multiplex families, the inheritance of BEEC would be consistent with autosomal dominant with reduced penetrance, autosomal recessive, or X-linked patterns [19]. The lack of recurrence may in part be due to reduced reproductive fitness. This may change due to surgical advances and improvements in reproductive medicine facilitating the birth of biological children to affected individuals. Studies have shown that individuals with CBE with non-consanguineous and non-affected parents have a recurrence risk among siblings from 0.3 to 2.3% [23,24]. The recurrence risk for offspring from affected parents is 1.4%. The risk of having a second affected child from non-consanguineous and non-affected parents shows an approximate 400-fold increase compared to the general population [23].

Reutter et al., 2007 [21] reported higher concordance rates in monozygotic twins (62%) compared to dizygotic twins (11%) with BEEC, supporting a genetic etiology. A number of reports have shown recurrence of CE within families [25]; an increased occurrence in conjoined and monozygotic twins [26,27,28,29,30,31,32]; concordant conjoined twins [33], and discordant dizygotic twins [6]. Xu et al., 2020 [34] reported CE in twins (*n* = 28) and triplets (*n* = 2), including monozygotic (*n* = 20), dizygotic (*n* = 3), trizygotic (*n* = 2), and unknown zygosity (*n* = 5). Of the CE anomalies within the 20 monozygotic twins, 9 were concordant and 11 were discordant. The higher incidence of CE in monozygotic twins compared to dizygotic twins could suggest a possible genetic contribution to the occurrence of these anomalies. Fullerton et al., 2017 [35] reported that approximately 14% of CE cases occurred in same-sex twins, which supported their hypothesis that the embryogenesis of CE could be related to errors in monozygotic splitting.

## 5. Molecular Genetics of BEEC

### Chromosomal Variants Identified in BEEC

There is an extensive history of successful disease gene discovery made through the characterization of individuals with chromosomal abnormalities. These chromosomal changes provided a shortcut to identify relevant chromosomal loci for positional cloning approaches before next generation sequencing techniques transformed disease gene discovery over the past decade. Translocations disrupting disease-associated genes and deletions harboring the causative gene led to some of the earliest disease gene identifications in the last century.

Cytogenetic analyses have identified a number of chromosomal anomalies in individuals with BEEC, summarized in Table 2. The higher prevalence of CBE and epispadias in males would be consistent with a sex chromosome linked etiology and a number of cases with sex chromosome aneuploidies have been reported, including 47, XXY [36] and 47, XYY [37]. Lin et al., 1993 [38] reported a child with CE associated with unilateral renal agenesis and Müllerian anomalies with a 47, XXX karyotype. Husmann and Vandersteen, 1999 [39] reported individuals with CE with 47, XXX and 45, X/46, XX mosaicism. Soderhall et al., 2014 [40] reported a rearrangement on the X chromosome consisting of a gain at Xq26.3-qter and a loss at Xp22.12-pter, including loss of one copy of the SHOX gene on Xp22.3. Despite these individual reports there is no compelling evidence to support a sex-linked genetic etiology.

Multiple autosomal chromosome anomalies have been reported in association with BEEC. Zaki et al., 2012 [41] identified a de novo 10.4 Mb deletion of chromosome 1qter in an Egyptian boy presenting classic features of chromosome 1q43q44 deletion syndrome with CBE: An absent phallus, extreme hypoplastic scrotum, and an anteriorly displaced anus.

Boyadjiev et al., 2005 [37] identified a male with CBE with a de novo translocation, 46,XY, t(8;9)(p11.2;q13). The chromosome 9q13 breakpoint maps to involve a single gene: contactin associated protein-like 3, (CNTNAP3), which belongs to the larger Neuroxin-IV/CNTNAP/Paranodin (NCP) family, which mediates neuron–glia interactions.

El-Hattab et al., 2010 [42] reported the first case of an infant with OEIS complex and a 2.4 Mb terminal deletion on chromosome 1p36. Monosomy 1p36 is the most common terminal deletion syndrome, and is characterized by typical facial features, developmental delay, and heart, skeletal, genitourinary, and neurological defects [43]. This deletion harbors approximately 70 genes, of which three have been postulated to have contributing roles to CE: NOC2L, which encodes an inhibitor of histone acetyltransferase that plays a role in transcriptional regulation [44]; DVL1, which acts as a mediator of the WNT signaling pathway [45,46,47], and MMP23B, which encodes a member of the matrix metalloproteinase family, that are involved in the breakdown of extracellular matrix in embryonic development and tissue remodeling [48].

Thauvin-Robinet et al., 2004 [49] reported a patient with CE and a de novo unbalanced translocation between the long arm of chromosome 9 and the long arm of chromosome Y, resulting in a 9q34.1-qter deletion. A further report by Kosaki et al., 2005 [50] reported a patient with CE with a de novo deletion at chromosome 3q12.2–3q13.2. However, among eight patients reported with an interstitial deletion involving 3q12–q13, this was the only individual with CE. Haploinsufficiency of these deletions alone may not be sufficient to result in CE, but it is possible that compound heterozygosity of a deletion with a rare variant on the other allele could lead to the phenotype.

A study undertaken by Harrison et al., 2014 [51] of 17 females with CE, using array comparative genomic hybridization, revealed copy number variants in seven (41%), comprising five gains (3p26.3, 12q14.2, 16p11.2, 16p13.2 and 21q22.3) and two losses. Two copy number variations were novel, including a paternally inherited duplication on 16p13.2 and a 7 Mb de novo deletion at 1q32. In a follow up study, the same group reported a heterozygous de novo 0.9 Mb microduplication on chromosome 19p13.12 in one CBE patient from a sample study of 110 patients [52]. It is difficult to distil from these data whether individuals with BEEC are more prone to chromosomal anomalies which contribute to the pathogenesis of the disorder, or an ascertainment bias in that these children undergo routine karyotyping. The former is likely, but has not yet resulted in a more precise definition of the specific mechanisms underlying the malformation spectrum. However, there is one chromosomal anomaly, 22q11 duplication, which has a clear association with BEEC.

## 6. Chromosome 22q11 Micro Duplications and BEEC

Draaken et al., 2010 [53] performed molecular karyotyping by SNP array analysis on 16 patients with non-syndromic BEEC. They identified a de novo microduplication of chromosome 22q11.21 in one patient with CBE. Subsequent multiplex ligation-dependent probe amplification (MLPA) analysis on a further 50 patients with non-syndromic BEEC identified an overlapping 22q11.21 duplication in one CBE patient, inherited from the unaffected mother.

From a cohort of 244 BEEC cases, Lundin et al., 2010 [54] reported two patients with a ~3 Mb microduplication involving 22q11.2. One was de novo, with the other inherited from the unaffected mother. Copy number variants at 22q11.2 are associated with a number of genomic disorders, including DiGeorge/velocardiofacial syndrome, cat-eye syndrome, and the der(22) syndrome. At the 22q11.2 locus, deletions are relatively common compared to duplications. We reported four patients with 22q11 duplications from a UK BEEC cohort [55]. This is consistent with data from other studies that identified 22q11 duplications in approximately 3% of cases (Table 3). This is the most strongly associated genetic variation with BEEC, conferring a twelve-to-thirty-fold increased risk [55,56]. It is possible that the origin of the allele (paternal or maternal) that the duplication has arisen on, or the parent from whom the duplication has been inherited, alters the risk of BEEC. However, incomplete data reporting and the small number of cases cannot confirm any specific relationship.

A comparison of eight previously reported 22q11.21 duplications in individuals with CBE revealed a 414 kb “phenocritical” region, encompassing 10 candidate protein coding genes [58]. Within this phenocritical region were five genes: CRKL; AIFM3; LZTR1; THAP7, and P2RX6, which were defined by refining the locus due to overlapping deletions and duplications, resulting in disorders of the lower urinary tract [57]. Notably, loss of function variants in CRKL are associated with congenital anomalies of the kidney [59]. Further work is required to define the specific gene or genes within this locus that contribute to BEEC pathogenesis.

## 7. Candidate Genes and Genome Wide Association Studies (GWAS) in BEEC

In contrast to the potential high penetrance effect of chromosomal changes, candidate and genome wide association studies (GWAS) investigate the contribution of more common variants, conferring a smaller effect size. Both association study approaches seek to genotype large cohorts of individuals with BEEC and compare allele frequencies of SNPs in these against ethnically matched controls.

To date, only a small number of candidate gene association studies have been undertaken, the first of those by Wilkins et al., 2012 [60] to investigate the contribution of TP63 to the formation of human BEEC. The group hypothesized that variants in TP63 are involved in the pathogenesis of BEEC. Sequencing of the deltaNp63 promotor region in 163 BEEC patients and 285 ethnicity matched controls identified seven single nucleotide polymorphisms and four insertion/deletion (indel) polymorphisms. These indel polymorphisms were associated with an increased risk of BEEC. The indel polymorphism sites significantly differed between Caucasian and non-Caucasian populations. A 12-base-pair deletion was associated with an increased risk in only Caucasian patients (*p* = 0.026, odds ratio (OR) = 18.33) whereas a four-base-pair insertion was associated with an increased risk in non-Caucasian patients OR = 4.58. Using luciferase assays, they showed a consistent statistically significant reduction in transcriptional efficiencies of the promotor sequences containing indel polymorphisms, suggesting that indel polymorphism of the deltaNp63 promoter leads to a reduction in p63 expression which could potentially lead to BEEC.

Reutter et al., 2014 [61] conducted a GWAS in 218 CBE cases, 865 controls, and 78 trios, all of European descent. They found suggestive evidence for association with CBE in a highly conserved 32 kb intergenic region containing regulatory elements between WNT3 and WNT9B, both of which have been associated with urogenital anomalies in humans and mice [62,63]. The strongest associated SNP in the region (rs9890413) resides ~4 kb next to the WNT3 promoter, a region highly conserved in amniotes. It is worth noting that in a study by Vlangos et al., 2011 [64], in a cohort of 13 patients with CE they identified variants within the 5′UTR region of WNT3. Nakamura et al., 2011 [65] identified a number of potential transcription factor-binding motifs that exist within the WNT3 promoter region, several of which have been found to be differentially expressed in human newborn bladder exstrophy tissue and are also important for the promotion of the embryonic urorectal septation process [66,67]. This region also contains regulatory elements which regulate Wnt signaling via p63 possibly suggesting that there are regulatory domains with the intergenic regions that can modulate Wnt signally via a conserved WNT3-WNT9B-p63 regulatory module in urorectal and urogenital development [68]. Korberg et al., 2015 [69] evaluated WNT-pathway genes in a cohort of 20 BEEC patients by parallel sequencing. They identified a de novo variant in the WNT3 gene in one patient (c. 271T>C, p. Cys91Arg). Further sequencing of WNT3 in a further 410 BEEC patients revealed a single additional variant (c.638G>A, p.Gly213Asp) which was paternally inherited. Knockdown of WNT3 in zebrafish revealed cloacal malformations, including disorganization of the cloacal epithelium and expansion of the cloacal lumen [69].

Draaken et al., 2015 [70] performed a GWAS in 110 patients with CBE and 1177 controls of European origin. An association with CBE was identified within a 220 kb region on chromosome 5q11.1, harboring the ISL1 (ISL LIM homeobox 1) gene. A meta-analysis was performed using the data from their previous GWAS of 98 CBE patients and 526 controls of European origin [61]; these data also implicated the 5q11.1 locus in CBE risk. In total 138 markers at this locus reached genome-wide significance. Murine expression analyses showed evidence of ISL1 expression in the genital regions, including peri-cloacal mesenchyme and the urorectal septum within the critical time frame for human CBE development. Following on from this study Zhang et al., 2017 [71] performed an GWAS in a cohort of 268 CBE patients of Australian, British, German, Italian, Spanish and Swedish origin and 1354 ethnically matched controls and from 92 CBE case parent trios from North America. Only one marker rs6874700 at the ISL1 locus showed significant association with CBE (*p* = 2.22 × 10^−8^). Meta-analysis of rs6874700 from this study and the previous study by Draaken et al., 2015 [70] showed compelling association (*p* = 9.2 × 10^−19^). Analysis of ISL1-expressing cells by a lineage tracer mouse model showed ISL1-expressing cells in mouse model in the urinary tract E10.5 and distributed in the bladder E15.5. In zebrafish larvae, staged 48 HPF ISL1 expression was detected in a small region of the developing pronephros. This association together with functional studies in mouse embryos and zebrafish larvae suggest ISL1 as an important susceptibility gene for CBE and as a regulator of urinary tract development.

## 8. Monogenic BEEC

The lack of large multiplex families has made traditional positional cloning approaches to disease gene discovery incredibly challenging in BEEC. Ludwig et al., 2009 [18] conducted a genome-wide linkage analysis in two pedigrees, Spanish and German, each having two family members affected with CBE. Parametric linkage analysis under a recessive model with complete penetrance identified seven loci across chromosomes 2, 4, 7, 14, and 19 with LOD scores >1.50. Reutter et al., 2010 [72] also conducted a genome-wide linkage in a consanguineous Iranian multiplex family with an affected sibling pair. The male sibling had CBE and the female sibling had epispadias. Here analysis identified seven loci with LOD scores >1.60. Haplotype analysis showed that the siblings were homozygous identical by descent for all seven loci. Two of these regions overlapped with regions previously observed, one on chromosome 4q31.21–22, and one on chromosome 19q13.31–4 [72].

The introduction of exome and genome sequencing technologies and large sequence variant databases in healthy controls have opened opportunities to determine the effects of rare variants that may be enriched in individuals with BEEC. Reutter et al., 2016 [73] were the first to perform whole exome sequencing in eight patients (and parents) with CE. A pathogenic de novo variant was identified in the SLC20A1 gene. Following on from this study, Rieke et al., 2020 [74] sequenced SLC20A1 in a cohort of 690 patients with BEEC together with 84 patients with CE. Two further de novo variants were identified. They investigated the functional role of SLC20A1 in urinary tract development using knockdown of SLC20ALA in zebrafish. This resulted in kidney cysts and malformations of the cloaca, and the morphants also demonstrated dysfunctional voiding and hindgut opening defects, mimicking the imperforate anus in human CE. Immunohistochemistry of a 6-week-old human embryo detected SLC20A1 in the urinary tract and the abdominal midline, structures implicated in the pathogenesis of CE. Results from this study suggest SLC20A1 is involved in human and zebrafish urinary tract and urorectal development and implicates SLC20A1 as a disease associated gene for BEEC.

## 9. Conclusions

The application of array-based, GWAS, and next generation sequencing techniques in large BEEC cohorts has helped to identify putative disease-causing genes and chromosomal regions in the human genome for both Mendelian and multifactorial BEEC. Functional analysis of embryonic pathways provides a better understanding of the molecular biological mechanisms underlying normal, urorectal, and genitourinary malformations within the embryology of the human urogenital system.

It is reasonable to propose that both inherited and de novo highly penetrant variants could be relevant to the etiology of BEEC as they have been shown for many genetically heterogeneous congenital birth defects such as congenital heart disease.

New approaches such as gene and pathway enrichment analyses of high-impact de novo variants from whole exome or whole genome data in parent-offspring trios will likely aid in the identification of novel genes and/or pathways to better understand the underlying genetic mechanisms of BEEC, and the potential to use these data to develop therapeutic approaches to help children affected by this devastating congenital disorder.

## Figures and Tables

**Table 1:** BEEC and associated birth defects adapted from Ludwig et al., 2009 [18].

Type	Type of BEEC	OMIM
**BEEC-associated syndromes**		
Al Awadi/Raas-Rothschild syndrome	CBE	276820
Acrorenal syndrome	CBE	102520
Duane’s syndrome	CBE	126800
Elis-van Creveld Syndrome	E	225500
Epidermolysis bullosa junctionalis	CBE	226650
Epstein syndrome	CE	153650
Fraser syndrome	Pseudoexstrophy	219000
Goldenhar syndrome	CE	164210
Goltz-Gorlin syndrome	CE	228250
Gollop-Wolfgang complex	CE	305600
Microcephalic osteodysplastic primordial dwarfism type III	CBE	210730
Oculoectodermal syndrome	CBE	600268
Opitz G/BBB syndrome	CBE	145410
**BEEC associations**		
Axial mesodermal dysplasia	CE	608160
Caudal dysplasia	CBE	600145
VATER association	CBE	192350
**BEEC-associated Malformations**		
***Head and neck***		
Chiari I malformation	CE	118420
Frontonasal dysplasia	CE	136760
Otocephaly-holoprosencephaly	CE	202650
Posterior cleft palate	CE	119540
Severe early-onset hearing loss	CE	561000
***Skeletal***		
Bilateral club feet	CE	119800
Severe lower limb defects	CE	-
Right thumb hypoplasia	CE	-
***Cardiovascular***		
Duplication of vena cava	CE	-
DORV, PV-atresia, right-sided aortic arch with PDA	Covered CBE	217095
***Abdomen***		
Gastroschisis	CBE	230750
Gastroschisis	Pseudoexstrophy	230750

BEEC, bladder-exstrophy-epispadias complex; CBE, classic bladder exstrophy; E; epispadias; CE, exstrophy of the cloaca; DORV, double outlet right ventricle; PV, pulmonic valve; PDA, patent ductus arteriosus.

**Table 2:** Chromosomal anomalies in patients with BEEC adapted from Ludwig et al., 2009 [18].

Chromosomal Location	Type of Mutation/Genotype	Phenotype
1q32.1q32.3	*De novo* 7.245 Mb deletion	PC
1q43q44	*De novo* 10.4 Mb deletion	CBE with absent phallus
	Diploid/Tetraploid/t(1; 6) mosaicism [in fibroblasts: 16% (3 cells) 92,XXXX;11% (2 cells) 46,XX,t(1; 6)(p32; q13); 73% (14 cells) 46,XX]	CE with hypo- melanosis of Ito
t(2;9)(q13;q32)	Translocation between 2q13 and 9q32 (46,XY)	CBE
3q12.2e13.2	*De novo* deletion (46,XY)	CE
4p	Deletion in the short arm of chromosome 4 (46,XY)	E
4p	46,XX,4p-	CBE
t(8;9)(p11.2;q13)	Translocation between 8p11.2 and 9q13 (46,XY)	CBE
9q34.1-qter Deletion	*De novo* unbalanced translocation between chromosome 9q and Yq. 46,Xder(Y)t(Y; 9)(q11.23; q34.1)edel(Y)(q11.2),der(9)t(Y; 9)	CE
9p	Duplication [dup(9p)] of the short arm (47,XY)	E
16p13.2	Paternally inherited duplication	PC
19p13.12	*De novo* 0.9 Mb microduplication	CBE
21	Duplication of chromosome 21 [dup(21)] (47,XX)	CE
Xq26.3->qter	Gain in region	CBE
Xp22.12->pter	Loss in region	CBE
	45,X0/46,XX mosaicism	CE
	47,XX, +21	CBE
	47,XY, +21	CBE
	47,XXX	CE
	47,XXY	E
	47,XYY	CBE
	Trisomy 18 (no sex reported)	CE

BEEC, bladder-exstrophy-epispadias complex; CBE, classic bladder exstrophy; E; epispadias; CE, cloacal exstrophy; PC, persistent cloaca.

**Table 3:** 22q11.21 duplications previously reported associated with BEEC.

Sex	BEEC Type and Associated Anomalies	Size of Duplication (Mb)	Segregation Pattern	References
Female	CBE	2.53–3.11	*De novo*	[54]
Male	CBE	2.51–2.86	Inherited from unaffected mother	[54]
Female	CBE, impaired hearing, and scoliosis	2.54–3.2	*De novo*	[55]
Female	CBE, impaired hearing, and mild neuropsychiatric disorder	2.7–3.3	Inherited from unaffected mother	[55]
Male	CBE	2.65–3.07	*De novo*	[57]
Male	CBE	0.67–1.26	Inherited from unaffected father	[57]
Male	CBE	0.35–0.62	*De novo*	[57]
Male	CBE	0.65–1.06	*De novo*	[57]
Male	CBE	~2.73	Inherited from unaffected mother	[56]
Male	Epispadias	~2.4	Inherited from unaffected mother	[56]
Patient 1	CBE, impaired hearing, and mild neuropsychiatric illness	~2.57	Inherited from unaffected mother	[58]
Patient 2	CBE, mild neuropsychiatric illness	~2.57	n.a. ^a^	[58]
Male	Glanular epispadia with dorsal curvation, duodenal atresia, single transverse palmar crease, high forehead, large eyes, protruding tongue	~2.57	*De novo*	[58]

^a^ Parental data were not available (n.a.).
